# The emotional state and social support of pregnant women attending childbirth classes in the context of physical activity

**DOI:** 10.1038/s41598-022-23971-7

**Published:** 2022-11-11

**Authors:** Joanna Kowalska, Małgorzata Dulnik, Zbigniew Guzek, Kinga Strojek

**Affiliations:** 1 Faculty of Physiotherapy, University of Health and Sport Sciences, Paderewskiego 35 Street, 51-612 Wrocław, Poland; 2 Ślęza Recreational Center, Błękitna 2, 55-040 Bielany Wrocławskie, Poland; 3 Department of Neurological Rehabilitation, University Hospital in Zielona Góra, 65‐046 Zielona Góra, Poland

**Keywords:** Psychology, Health care

## Abstract

Childbirth classes combined with elements of psychoprophylaxis and psychoeducation prepare women and their partners for childbirth and influence the level of their perceived stress and mood disorders. Participation in these classes may come as a form of support for pregnant women, or an opportunity to build self-efficacy and commence regular physical activity. The aim of this study was assess the emotional state, social support and self-efficacy of pregnant women attending childbirth classes, in the context of physical activity undertaken. The study included 101 pregnant women. The Berlin Social Support Scale (BSSS), the General Self-Efficacy Scale (GSES), the Perceived Stress Scale (PSS-10) and the State-Trait Anxiety Inventory (STAI) were used during their first day of attendance at the childbirth classes (initial survey; T1) and after 10 weeks of participation in these classes (final survey; T2). After 10 weeks of childbirth classes, there was a statistically significant change in the BSSS, specifically in the area of Perceived Available Support, GSES, PSS-10, and STAI X-2. There was no significant relationship established between the emotional state and the physical activity undertaken before and during pregnancy or with the social support received.

## Introduction

Pregnancy is often considered a stressful situation, a so-called critical incident. The emotional impact of pregnancy may change a woman’s functioning inside and outside the home. It can create a change in relation to the world as well as to oneself^1,2^. Pregnancy and birth of a child is one of the most important events in a woman's life, where a woman may experience increased level of perceived stress and anxiety, as well as symptoms of depression. This affects both the physical and mental state of a woman^3–6^. Unfortunately, poor mental state may contribute to complications during pregnancy and delivery (e.g. preterm labor)^5,6^. The consequences of woman's poor mental state also affect the child's health (e.g. low birth weight)^7,8^.

Many of factors influence the emotional state of pregnant women, including social support^3^. Social support is a voluntary act of a person (giver) for the benefit of another person (receiver). The recipient's reaction to this is positive and may be immediate or delayed^9,10^. The provider of this support may be a family member, partner, friend, or another person or institution, and the support may come in different forms (emotional, physical, instrumental, informational, etc.)^10,11^. Research by de Castro et al. confirms that social support is a protective factor, that prevents postpartum depression, especially in adolescent mothers^12^. Other research emphasizes an association between the presence of depression and anxiety before childbirth and poor marital ties, episode of mental illness, negative attitude towards pregnancy and lack of social support, increasing the risk of postpartum depression^13–15^. Lack of social support is also a risk factor for depression during the pregnancy^14^.

Perceived self-efficacy also appears to be important during pregnancy and preparations for childbirth. A higher sense of self-efficacy increases the motivation to act, even in the face of increased failures and troubles, and it is associated with better individual achievements (both in personal and professional life). In contrast, low self-efficacy is associated with high level of stress, depressive symptoms, anxiety and helplessness^16–18^.

Furthermore, according to research, appropriately selected physical exercise (recommended in childbirth classes) and physical activity during pregnancy help prepare women and their bodies for delivery, reducing the risk of depression^19,20^. The influence of exercise on depression, based on the psychological ("self-belief" and the distractor concept) and biological theories (including β-endorphin and thermogenic theory) are particularly important^21,22^.

The WHO and the American College of Obstetricians and Gynecologists recommend 150 min of moderate-intensity exercise per week both during pregnancy and after delivery. Additionally, women who performed high-intensity aerobic exercises or were physically active on a daily basis prior to pregnancy should continue these activities during pregnancy and postpartum^21,23^.

Childbirth classes combined with elements of childbirth psychoprophylaxis and psychoeducation prepare women and their partners for childbirth and influence the level of their perceived stress and mood disorders. Participation in these classes may come as a form of support for pregnant women, or an opportunity to build self-efficacy and commence regular physical activity^24^.

The aim of this study was therefore to assess the emotional state, social support received and self-efficacy of pregnant women attending childbirth classes, in the context of physical activity undertaken. The research questions guiding the study were: Is there a relationship between emotional state and physical activity undertaken both before and during the pregnancy and the social support received? Does participation in childbirth classes positively affect women’s emotional state and sense of self-efficacy? The two research hypotheses were assumed: There is a relationship between the emotional state of pregnant women and their physical activity and the social support. Participation in childbirth classes is associated with the improvement of the emotional state of pregnant women and an increase their self-efficacy.

## Materials and methods

### Study sample

The study was conducted during childbirth classes undertaken at the University Clinical Hospital in Wroclaw.

The attending gynecologist proposed the possibility of participation in the childbirth classes. The study included 152 pregnant women enrolled in the childbirth classes, who met the following inclusion criteria: informed consent to participate in the study, 21–31 weeks of gestation, consent of the attending physician, no contraindications to participation in childbirth classes, and physiologic pregnancy. The exclusion criteria were: past childbirth and previous pregnancies ending in miscarriage. The above inclusion and exclusion criteria resulted from the admission requirements of childbirth classes and the minimization of the influence of emotions resulting from previous experiences related to another pregnancy or miscarriage.

**Figure 1:**
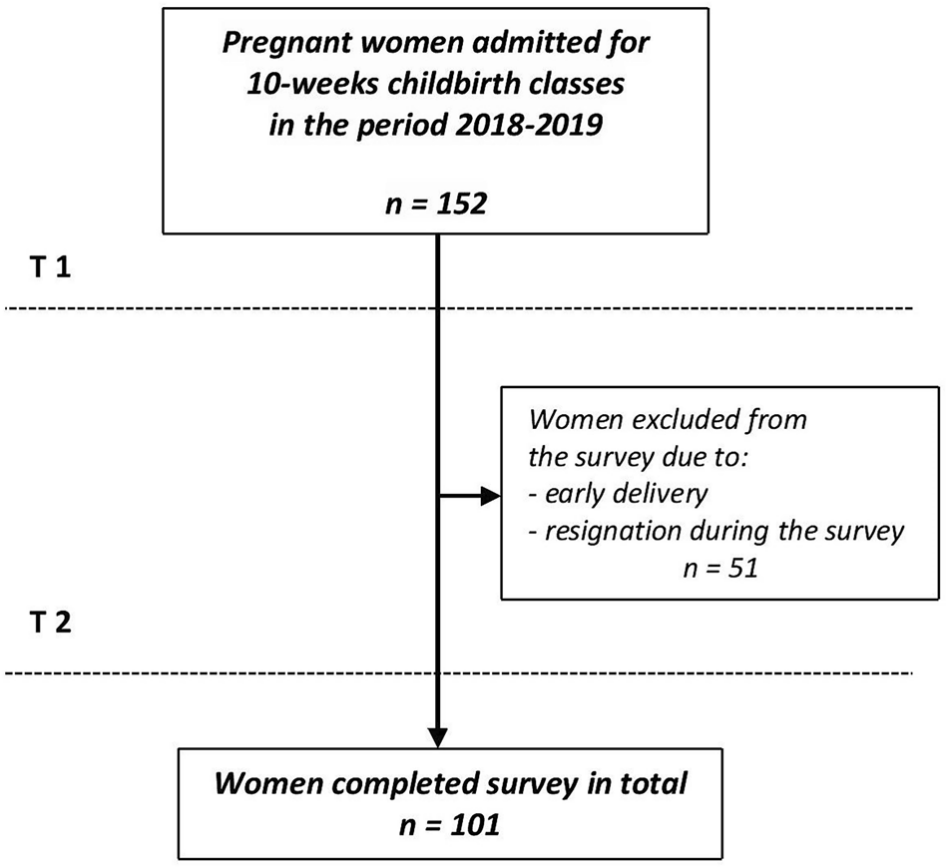
Recruitment process for the study sample.

A total of 101 women completed the study. The reduction in sample size was due to some women dropping out during the study, not attending childbirth classes, and early delivery (Fig. 1).

Only complete surveys were used for statistical analysis. Results were obtained in the initial and in the final survey (following 10 weeks of participation in childbirth classes).

Detailed characteristics of the study sample are presented in Table 1.

**Table 1:** Characteristics of the study sample, N = 101.

Feature	Mean (SD) or N (%)
**Age [years]**	
Mean (SD)	30.4 (3.6)
Range	23–43
**Week of pregnancy**	
Mean (SD)	27.2 (2.9)
Range	21–31
**Marriage length [years]**	
Mean	2.5 (1.9)
Range	0.5–10
**Education**	
Secondary	10 (9.9)
Higher	91 (90.1)
**Marital status**	
Lonely	23 (22.8)
In a relationship	78 (77.2)
**Place of residence**	
Village	7 (7.0)
City	94 (93.0)
**Type of employment**	
Employment contract	86 (85.1)
Contract of mandate	3 (3)
Other	12 (11.9)
**Perceived stress before childbirth**	
Yes	76 (75.2)
No	25 (24.8)
**The presence of an accompanying person during childbirth classes**	
Yes	81 (80.0)
No	20 (20.0)
**Physical activity before pregnancy**	
Yes	81 (80.0)
No	20 (20.0)
**Physical activity during pregnancy**	
Yes	59 (58.4)
No	42 (41.6)
**Frequency of physical activity during pregnancy [days a week]**	
Mean	3.0 (1.8)
Range	1–7
**Planned family birth**	
Yes	71 (70.0)
No/I do not know	30 (30.0)

### Methods

The Polish adaptations of well-known and common scales with good psychometric properties were used: the Berlin Social Support Scale (BSSS), the General Self-Efficacy Scale (GSES), the Perceived Stress Scale (PSS-10) and the State-Trait Anxiety Inventory (STAI). Additionally, a self-administered questionnaire was used. The self-administered questionnaire included basic socio-demographic data (i.e. age, gender, occupation, marital status and questions related to pregnancy, planned delivery and physical activity undertaken).

The Polish version of the BSSS adapted by Łuszczyńska et al. consists of 32 statements divided into 4 main areas: perceived availability of support (8 statements), need for support (4 statements), seeking support (5 statements) and currently receiving support (15 statements). For each of the statements, the respondent marks numbers from 1 to 4, where 1 means the statement is completely false, 2—slightly true, 3—moderately true, 4—completely true. The more points, the higher the sense of social support. The BSSS has satisfactory psychometric properties (Cronbach’s alpha ranges from 0.71 to 0.90)^25^.

The General Self-Efficacy Scale (GSES), in Juczyński's Polish adaptation, measures the strength of the individual's general belief in the effectiveness of coping with challenging situations and difficulties. It consists of 10 statements. The respondent marks numbers from 1 to 4, where 1 means: no, 2—rather not, 3—rather yes, 4—yes. The sum of points corresponds to the indicator of self-efficacy. The more points, the higher the feeling of self-efficacy. Cronbach's alpha was 0.85^26^.

The Polish adaptation of the PSS-10 by Juczyński and Ogińska-Bulik consists of 10 questions and measures subjective feelings related to stressful situations. The higher the score (maximum 40 points) the higher the intensity of perceived stress. The 0–13 points mean low score, 14–19 points—average score, and 20–40 points—high score. The Cronbach's alpha was 0.86^27^.

The STAI is used to evaluate the anxiety as a state (STAI X-1) and anxiety as a trait (STAI X-2). The STAI X-1 consists of 20 responses. The respondent selects one of four responses that describes how they feel at that particular moment (1—definitely no; 4—definitely yes). The STAI X-2 consists of 20 responses where the respondent selects one of four responses that describes how they generally feel (1—almost never; 4—almost always). The score achieved indicates the level of anxiety, where the higher the score, the higher the level of anxiety. The threshold for dividing respondents into subgroups with low and high levels of anxiety for the STAI X-1 was a score of 44, and for the STAI X-2, a score of 46 was used. The Cronbach’s alpha was 0.89 for STAI X-1 and 0.83 for STAI X-2)^28^.

### Procedure

The study was conducted in accordance with the Helsinki Declaration. Approval was obtained from the Bioethics Committee of the University of Health and Sport Sciences in Wroclaw, Poland (reference no. 40/2018).

The women who enrolled in the study were surveyed during their first day of attendance at the childbirth classes (initial survey; T1) and after 10 weeks of participation in these classes (final survey; T2).

During the 10 weeks of childbirth classes, the women participated in theoretical and practical courses. During the theoretical courses, they learned about the physiology of pregnancy, the process of labor, ways to relieve labor pain, and received information on puerperium. During practical training, the women learned breathing techniques used during childbirth and prepared themselves for caring for a newborn baby. Physical activity was also an important part of the childbirth classes schedule. This activity was aimed at maintaining general fitness, improving efficiency and preparing the future mother for the next stages of pregnancy and delivery. In addition to proper breathing techniques, much emphasis was placed on learning to push, as well as relaxation exercises and positions to reduce pain in the lumbar-sacral region. The training was held under the supervision of a qualified childbirth classes instructor (midwife, physiotherapist, gynecologist).

### Data analysis

In order to describe the results, the following descriptive statistics were used: mean, standard deviation, median and inter-quartile range (IQR) and in the case of qualitative variables percentages and numbers. Normality of distribution was verified using the Kolmogorov–Smirnov test. The normality of distribution has not been confirmed (except the BSSS); so in order to compare the initial and final tests, the Wicoxon's test was used. To measure the BSSS, the Student's test was used. Due to the small size of subgroups, non-parametric tests were used for statistical analysis, despite the normal distribution (as in the case of BSSS). To assess the significance of differences between subgroups (activity women before pregnancy vs non activity women before pregnancy; activity women during pregnancy vs non activity women during pregnancy), the Mann–Whitney U test was used. Additionally, to determine the quantity of the effect of differences between examined groups, a Cohen’s d test was used. The strength of the correlation between the selected pairs of variables was assessed using Spearman correlations. Statistical tests were verified at a significance level of p < 0.05.

## Results

**Figure 2:**
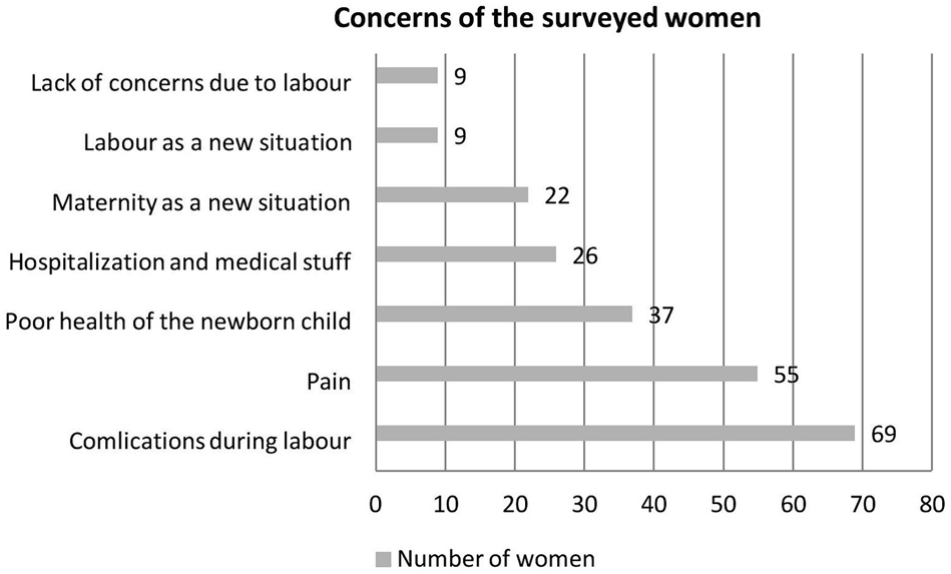
Concerns of the surveyed women.

Responses to the self-administered questionnaire demonstrated that pregnant women were most concerned about labor complications (68.3%) and labor pain (54.5%). Detailed data is shown in Fig. 2.

In the initial survey (T1), as many as 82 (80.4%) women were found to have high level of stress (PSS-10). No score indicating low level of stress was reported. After the 10-week childbirth classes (T2), the number of women with high stress level decreased to 65 (63.7%). One woman had a score indicating a low stress level, while 35 (34.3%) of the participants had an average stress level.

As many as 90 (88.2%) of the participants had scores indicative of high anxiety as a state (STAI X-1) at the initial survey (T1) and 85 (83.3%) participants at the final survey (T2).

In contrast, for anxiety as a trait (STAI X-2), 65 (63.7%) of the participants achieved high level at T1, while at T2, a total of 52 (51%) of the participants achieved such results.

After 10 weeks of childbirth classes (T2), there was a statistically significant change in the BSSS, specifically in the area of Perceived Available Support, GSES, PSS-10, and STAI X-2 (Table 2).

**Table 2:** The results of the BSSS (Wilcoxon test), GSES, PSS-10 and STAI (t-Student’s test) at initial (T1) and final (T2) studies.

Scales	T1		T2		Test t-Student’s/Willcoxon test		Effect size
	Mean (SD)	Median (IQR)	Mean (SD)	Median (IQR)	t/Z	p	Cohen’s d
BSSS	103.6 (8.3)	106.0 (10.0)	103.8 (8.8)	105.0 (10.0)	− 0.75	0.4532	0.0241
I. Perceived availability of support	29.6 (2.8)	31.0 (4.0)	30.1 (2.6)	31.0 (2.5)	− 2.50	0.0124*	0.1785
II. Need for support	11.3 (1.7)	12.0 (3.0)	11.1 (1.7)	11.0 (2.0)	− 0.89	0.3681	0.1176
III. Seeking support	14.6 (2.9)	15.0 (4.0)	14.5 (2.9)	15.0 (4.0)	− 1.06	0.2891	0.0345
IV. Currently receiving support	48.0 (4.2)	50.0 (5.0)	48.1 (5.1)	50.0 (4.0)	− 1.29	0.1971	0.0238
GSES	30.6 (3.9)	30.0 (5.0)	32.2 (3.9)	32.0 (5.0)	4.50	< 0.0001*	0.4102
PSS-10	35.7 (7.1)	22.0 (4.0)	33.2 (5.6)	21.0 (5.0)	− 3.29	0.0013*	0.3521
STAI X-1	44.5 (3.9)	48.0 (4.5)	47.9 (5.6)	48.0 (5.0)	0.46	0.6413	0.8717
STAI X-2	47.0 (2.8)	47.0 (6.0)	45.7 (0.7)	46.0 (7.0)	− 3.43	0.0008*	0.4643

BSSS, Berlin Social Support Scales; GSES, General Self Efficacy Scale; PSS-10, Perceived Stress Scale; STAI X-1, The State-Trait Anxiety Inventory as a state; STAI X-2, The State-Trait Anxiety Inventory as a trait;T1, Initial results; T2, Final results; *p < 0.05.

When comparing the emotional state of the participants, there were no statistically significant differences found between the groups of physically active and inactive women during pregnancy (Table 3).

**Table 3:** The results of the BSSS, GSES, PSS-10 and STAI in physically active and physically inactive group of women during pregnancy (Mann–Whitney U test).

Scales	Physical activity during pregnancy					
	Yes; n = 59		No; n = 42		p	Effect size Cohen’s d
	Mean SD	Median IQR	Mean SD	Median IQR		
BSSS (T1)	104.2 7.9	106.0 10.0	104.4 8.1	106.0 11.0	0.9522	0.0250
BSSS (T2)	104.4 7.9	105.0 12.0	104.1 8.3	105.0 12.0	0.8572	0.0379
I. Perceived availability of support (T1)	29.9 2.4	31.0 4.0	29.9 2.3	31.0 3.25	0.9681	0.0000
I. Perceived availability of support (T2)	30.2 2.6	31.0 2.0	30.0 2.6	31.0 2.5	0.6171	0.0769
II. Need for support (T1)	11.3 1.7	12.0 3.0	11.3 1.8	11.5 3.0	0.98404	0.0000
II. Need for support (T2)	11.1 1.7	11.0 3.0	10.9 1.8	11.0 2.0	0.70394	0.2284
III. Seeking support (T1)	14.8 2.9	15.0 3.0	15.0 2.6	15.0 5.0	0.8572	0.0726
III. Seeking support (T2)	14.6 2.7	15.0 4.0	14.5 2.5	15.0 3.25	0.8493	0.0384
IV. Currently receiving support (T1)	48.2 4.1	50.0 4.0	48.2 4.0	50.0 5.0	0.9840	0.0000
IV. Currently receiving support (T2)	48.5 4.8	51.0 4.0	48.5 4.8	51.0 3.25	0.9124	0.0000
GSES (T1)	31.1 3.9	31.0 5.0	31.2 3.8	31.5 4.0	0.7039	0.0259
GSES (T2)	31.8 3.9	32.0 5.0	31.2 3.9	31.0 4.0	0.4533	0.1538
PSS-10 (T1)	22.2 3.8	22.0 5.0	22.3 4.1	22.0 5.0	0.8026	0.0253
PSS-10 (T2)	21.4 3.8	21.0 4.0	22.0 4.0	22.0 4.25	0.4777	0.1538
STAI X-1 (T1)	47.8 4.2	48.0 5.0	47.9 4.0	48.0 5.0	0.9840	0.0244
STAI X-1 (T2)	49.1 3.6	49.0 5.0	49.0 3.3	49.0 4.25	0.7414	0.0289
STAI X-2 (T1)	46.5 4.4	47.0 5.0	46.3 4.8	46.5 4.5	0.6745	0.0434
STAI X-2 (T2)	46.3 4.2	47.0 6.0	46.0 4.5	46.0 7.0	0.6672	0.0689

BSSS, Berlin Social Support Scales; GSES, General Self Efficacy Scale; PSS-10, Perceived Stress Scale; STAI X-1, The State-Trait Anxiety Inventory as a state; STAI X-2, The State-Trait Anxiety Inventory as a trait; T1, Initial results; T2, Final results; *p < 0.05.

A statistically significant difference was recorded in the GSES at T1 between the physically active and physically inactive group of women before pregnancy (Table 4).

**Table 4:** The results of the BSSS, GSES, PSS-10 and STAI in physically active and physically inactive group of women before pregnancy (Mann–Whitney U test).

Scales	Physical activity before pregnancy					
	Yes, n = 81		No, n = 20		p	Effect size Cohen’s d
	Mean SD	Median IQR	Mean SD	Median IQR		
BSSS (T1)	103.7 8.6	106.0 11.0	104.8 7.0	105.5 5.75	0.7795	0.1403
BSSS (T2)	103.3 9.3	105.0 12.0	103.6 7.9	105.0 12.5	0.9761	0.0348
I. Perceived availability of support (T1)	29.7 2.8	31.0 4.0	30.3 2.1	31.0 3.0	0.5287	0.2424
I. Perceived availability of support (T2)	30.0 2.8	31.0 3.0	30.3 2.1	31.0 2.0	0.9920	0.1212
II. Need for support (T1)	11.2 1.8	12.0 3.0	11.0 1.9	11.0 3.0	0.6891	0.1081
II. Need for support (T2)	11.0 1.7	11.0 2.0	10.7 1.6	11.0 2.0	0.5093	0.1817
III. Seeking support (T1)	14.8 2.9	15.0 3.5	15.1 2.8	15.0 4.25	0.6101	0.1052
III. Seeking support (T2)	14.5 2.8	15.0 4.0	14.4 2.6	14.5 3.75	0.9124	0.0370
IV. Currently receiving support (T1)	47.9 4.3	50.0 4.5	48.4 3.1	49.5 4.0	0.8807	0.1334
IV. Currently receiving support (T2)	47.8 5.5	50.0 5.0	48.1 4.5	50.0 3.0	0.9124	0.0597
GSES (T1)	30.7 4.0	30.0 5.0	32.4 2.3	32.5 3.75	0. 0455*	0.5210
GSES (T2)	31.9 4.0	31.0 5.5	31.4 3.6	31.5	0.8729	0.1314
PSS-10 (T1)	21.9 3.5	22.0 4.0	22.5 4.1	22.0 4.0	0.3576	0.1574
PSS-10 (T2)	21.0 3.6	21.0 4.0	22.2 4.4	22.5 5.0	0.1285	0.2985
STAI X-1 (T1)	47.7 4.2	48.0 5.0	48.7 3.6	48.5 5.0	0.4533	0.2556
STAI X-1 (T2)	48.6 3.5	49.0 5.0	49.6 3.5	49.5 5.5	0.2585	0.2857
STAI X-2 (T1)	46.6 4.6	47.0 5.0	46.5 4.3	47.0 3.75	0.8729	0.0224
STAI X-2 (T2)	45.7 4.7	46.0 7.0	45.3 4.8	44.5 6.5	0.6745	0.0842

BSSS, Berlin Social Support Scales; GSES, General Self Efficacy Scale; PSS-10, Perceived Stress Scale; STAI X-1, The State-Trait Anxiety Inventory as a state; STAI X-2, The State-Trait Anxiety Inventory as a trait;T1, Initial results; T2, Final results; *p < 0.05.

No statistically significant differences were obtained when comparing the emotional state of women with an accompanying person/a partner during childbirth classes and women without an accompanying person/a partner. Only self-efficacy at T1 was statistically significantly higher in the group of women who attended the childbirth classes without an accompanying person/a partner (p = 0.0498).

A statistically significant positive correlation was found between the STAI X-1(T2) and the BSSS(T1), the GSES (T1). Additionally, a significant correlation was observed between the STAI X-2(T1) and PSS-10 (T1 and T2); and between the STAI X-2(T2) and PSS-10(T2) (Table 5).

**Table 5:** Spearman’s correlation analysis for selected pairs.

rho	BSSS T1	BSSS T2	GSES T1	GSES T2	PSS-10 T1	PSS-10 T2	STAI X-1 T1	STAI X-1 T2	STAI X-2 T1
BSSS T2	0.69*								
GSES T1	0.10	0.13							
GSES T2	0.11	0.16	0.58*						
PSS-10 T1	0.07	0.01	0.12	0.11					
PSS-10 T2	0.14	0.01	0.10	-0.19	0.65*				
STAI X-1 T1	0.05	0.05	0.06	0.03	0.02	0.04			
STAI X-1 T2	0.20*	0.03	0.22*	0.11	− 0.06	0.05	0.38*		
STAI X-2 T1	0.14	0.18	0.19	− 0.03	0.23*	0.30*	0.05	0.01	
STAI X-2 T2	0.17	0.15	− 0.13	− 0.03	0.13	0.23*	0.13	0.11	0.06

BSSS, Berlin Social Support Scales; GSES, General Self Efficacy Scale; PSS-10, Perceived Stress Scale; STAI X-1, The State-Trait Anxiety Inventory as a state; STAI X-2, The State-Trait Anxiety Inventory as a trait; T1, Initial results; T2, Final results; rho—Spearman’s correlation coefficient; *p < 0.05.

## Discussion

Caring for not just the physical but also the emotional state of women is very important, particularly during pregnancy. Childbirth classes should ideally prepare a women and their partners for childbirth and parenthood. Previous studies have indicated a high effectiveness of such clasess. The results presented by Kowalska et al. demonstrated a lower level of stress in pregnant women attending childbirth classes in comparison to those who did not attend them^20^. A study by Bączyk et al. found a lower level of stress during the puerperium in women attending childbirth classes^29^.

The results of the present study also suggest a significant impact of childbirth classes, particularly in the area of social support, like the increase in perceived available support, increased level of self-efficacy and reduced stress and anxiety. Similar results were obtained by Stangret et al. who showed that the course participants were characterized by lower level of anxiety^30^. On the other hand, Piziak emphasized that the well-being of women who acquired information from childbirth classes was better compared to women who sought it on their own. Similar conclusions were presented by Augustyniak et al.^31,32^.

An important aspect of childbirth classes is their group form (not individual). The women, together with accompanying persons (most often partners, husbands), can complement their knowledge and skills, as well as establish new relations with the other participants during the course. This can build a higher sense of social support. A well-trained and qualified medical staff who conduct these classes also provides a sense of comfort and security.

Research by Rashid et al. reported that depressive symptoms in pregnancy are associated with poor social support^33^. Other researchers have also indicated the importance of social support for pregnant women, while the lacking of this support has been classified as a risk factor for depression and its symptoms^34,35^. Furthermore, Maliszewka et al. have emphasized that social support is important after the delivery. Breastfeeding in the initial period, together with satisfactory social support, have been found to reduce the risk of postpartum depressive disorders^36^.

It is worth noting that, in the study sample, the majority of women declared regular physical activity before pregnancy, and more than half during pregnancy. The most frequently chosen forms of activity were gymnastics, walking, Pilates and yoga. This was similar to the results of study by Ćwiek et al.^37^.

Although the present results did not demonstrate significant differences in the emotional state of the physically active and physically inactive women during pregnancy, some emerging trends that are consistent with the results of other research can be observed^38^. In the group of women who were active during pregnancy, stress level decreased and self-efficacy increased after 10 weeks. However, in both groups, there was an increase in anxiety as a state, which shows high tension and anxiety before the impending delivery date. This is in line with the fears reported in the surveyed group of women. Among the most frequently cited fears were complications related to childbirth and pain during the delivery. Similar results concerning the most common fears of pregnant women were reported by Kowalska et al. and Bączyk et al.^20,29^. Resarch by Dudziak et al. shows that women who are physically active during pregnancy are more confident in their ability to control labour pain. On the other hand, physically inactive women do not believe that they can influence the level of pain themselves and that it depends on intervention from medical staff or random factors^39^.

Other researchers emphasize the long-term impact of physical activity during pregnancy. Vargas-Terrones et al. and Kowalska et al. have reported a statistically significant difference in the incidence of postpartum depression in women who were active during pregnancy^19,20^. Different results were reported by De Vargas Nunes Coll et al., where physical activity in the group of pregnant women had not significantly reduced the incidence of postpartum depressive symptoms^40^. In a study by Fournier et al., it was noted that labour lasted shorter if women attended classes under a supervision of qualified staff, compared to pregnant women who exercised alone at home^41^.

When analyzing the uptake of physical activity before pregnancy, it can be noted that physically inactive women had significantly higher self-efficacy at the start of the classes, compared to physically active women. Interestingly, after 10 weeks of childbirth classes, the level of self-efficacy has decreased in group of inactive women, while in the group of women active before pregnancy, it increased (but not statistically significantly). It is worth noting that a significantly higher sense of self-efficacy was observed at first measurement point among unaccompanied women participating in childbirth classes, than in the group of women who were accompanied by a close person. Such results may suggest that women without support from a close person is more mobilized to prepare themselves as well as possible for childbirth and motherhood. On the other hand, a high sense of self-efficacy may cause a woman to decide that she does not need the support of a close person during the childbirth classes. In a study by Rogala and Ossowski, the relationship between the sense of self-efficacy and selected aspects of childbirth were analysed. The researchers observed that pregnant women with a normal course of pregnancy and delivery showed higher level of self-efficacy^42^.

Stress level followed a similar pattern. After 10 weeks, stress decreased in the group of women who were active before pregnancy, in contrast to women who were not physically active before pregnancy. When studying a group of women and men who had accompanied them, Kościcka et al. demonstrated lower level of general and intrapsychic stress in the group which was physically active^38^.

A higher self-efficacy improves persistence in action, health behaviors, engagement, or openness to action, and lowers stress level^16,43,44^. In the present study, correlation analysis did not reveal a strong relationship between the level of self-efficacy and the level of perceived stress. However, a higher level of self-efficacy was associated with a higher level of anxiety as a state, as evidenced by strong emotions accompanying the impending delivery. Additionally, it is worth mentioning that such a relationship was observed among pregnant women unaccompanied to childbirth classes and in the group of women who were physically inactive before pregnancy. In these groups, level of self-efficacy decreased at 10 weeks while the level of anxiety increased. Regardless of the magnitude of one's self-efficacy, the emerging fear of delivery was very strong. These findings may suggest a direction for prevention efforts in childbirth classes. In light of the upcoming birth, all these factors are significant and can benefit the course of child birth, to include the very aspect of experiencing this important event.

### Limitations and future perspectives

The present study had several limitations. The performed tests were screening in nature and are not related to the diagnosis (e.g., anxiety disorders or anxiety as a symptom of mood disorders). The tests were performed in one childbirth class; therefore, the results presented should not be generalized. The study should be replicated in order to increase the number of examined groups and subsequently subgroups. Moreover, the observation time could be prolonged up to the delivery and puerperium.

This study may lead to a reflection on the childbirth classes and the physical and emotional preparation of women for the upcoming events, such as childbirth, puerperium, and future parenthood.

## Conclusions

Following the 10 weeks of childbirth classes, the women participating in this research were characterized by significantly higher level of self-efficacy and perceived available support, as well as lower level of perceived stress and anxiety. This appears to be extremely important, especially in the context of mental health prophylaxis during pregnancy, the physical activities undertaken, and the preparation for childbirth. In the study sample, there was no significant relationship between the emotional state and the physical activity undertaken before and during pregnancy or with the social support received. Continuing the research is thus recommended, taking into account the women and their partners who attend childbirth classes.

## Data Availability

Te datasets used and/or analysed during the current study available from the corresponding author on reasonable request.
